# The Influence of Including Goalkeepers on the Intensity Demands of Walking Football Practice

**DOI:** 10.3390/sports12120346

**Published:** 2024-12-16

**Authors:** Júlio A. Costa, César André Coelho, António Ferraz, João Brito, José Guilherme, André Seabra, Bruno Travassos, Hugo Folgado, Bruno Gonçalves

**Affiliations:** 1 Portugal Football School, Portuguese Football Federation, FPF, 1495-433 Oeiras, Portugal; cesar.andre.coelho@gmail.com (C.A.C.); joao.brito@fpf.pt (J.B.); jose.guilherme@fpf.pt (J.G.); andre.seabra@fpf.pt (A.S.); bfrt@ubi.pt (B.T.); bgoncalves@uevora.pt (B.G.); 2 CIFD, Sports Research, and Training Center, Jean Piaget University of Angola, Luanda 2177, Angola; antferraz@hotmail.com; 3 Department of Sport Sciences, CIDESD, Research Center in Sports Sciences, Health Sciences, and Human Development, University of Beira Interior, 6201-001 Covilhã, Portugal; 4 KinesioLab—Research Unit in Human Movement, Institute of Piaget, 2805-059 Almada, Portugal; 5 Centre of Research, Education, Innovation and Intervention in Sport, Faculty of Sport, University of Porto, 4200-450 Porto, Portugal; 6 Departamento de Desporto e Saúde, Escola de Saúde e Desenvolvimento Humano, Universidade de Évora, 7004-516 Évora, Portugal; hfolgado@uevora.pt; 7 Comprehensive Health Research Centre (CHRC), Universidade de Évora, 7004-516 Évora, Portugal

**Keywords:** aging, soccer, physical activity, exercise, exploratory study

## Abstract

This preliminary study examined the effects of playing walking football with and without a goalkeeper (GK) on physiological, physical, technical, and perceptual variables in older men. Twenty participants (67 ± 4.7 years) engaged in two five vs. five walking football sessions, one WITH-GK and one WITHOUT-GK, using a randomized crossover design. The heart rate (HR), distance covered, technical actions, perceived exercise intensity, and enjoyment were measured. The results showed a significantly higher average HR (131 bpm vs. 123 bpm, *p* < 0.001), %HRmax (79% vs. 74%, *p* < 0.001), and time in higher HR zones (>80%HRmax, *p* < 0.05) in the WITH-GK condition. Participants also covered more total distance in the WITH-GK condition (1123 m vs. 1083 m, *p* < 0.001), particularly at speeds above 4 km/h (834 m vs. 781 m, *p* < 0.001). Conversely, more passes were made in the WITHOUT-GK condition (20 vs. 16 passes, *p* < 0.001), while the WITH-GK condition showed more shots (two vs. one, *p* < 0.001). The perceived exercise intensity was slightly higher in the WITHOUT-GK condition (five vs. four, *p* = 0.01), although the enjoyment levels were similar, with a slight preference for WITHOUT-GK. Overall, playing WITHOUT-GK provides a safer, lower-intensity alternative to WITH-GK, reducing the physical and perceived strain while maintaining enjoyment. This makes WITHOUT-GK particularly suitable for older adults or individuals with health conditions, promoting participation and rehabilitation with minimized risks.

## 1. Introduction

Walking football has become a promising alternative to traditional football, tailored to promote health benefits through older male adults’ interaction [1,2]. Emphasizing walking over running, walking football may mitigate injury risks while maintaining many tactical and technical elements of conventional football, promoting sustained physical engagement and social interaction [1,2]. Numerous studies have shown that structured activities, such as walking football, are essential for sustaining physical fitness, cognitive engagement, and social bonds, making them ideal for older populations [1,3,4,5]. The growth of walking football among the elderly reflects its alignment with accessible exercise modes, where physical exertion is balanced with strategic play and limited contact to suit older or mobility-restricted individuals, regardless of gender [1,2,5].

Research suggests that walking football provides considerable cardiorespiratory and metabolic benefits for middle-aged to elder individuals, while responses may vary depending on the game intensity and player familiarity with the sport [6,7]. Other studies indicate the potential for practice adjustments to affect outcomes among players with medical conditions. For example, Arnold, Bruce-Low and Sammut [3] show significant decreases in the body fat mass (11%) and BMI (3%) after a 12-week walking football program. Although research on chronic adaptations to walking football is limited, evidence suggests that sustained, regular participation in walking football with careful adjustment of the game intensity and duration might result in cardiovascular, muscular, and metabolic health benefits, emphasizing its value as an adjustable and accessible exercise for older adults [4].

Given the slower pace, modifications to the way walking football can be played, such as including or not a goalkeeper (GK), are hypothesized to impact the intensity and technical demands of walking football practice. Game configurations like these can potentially influence physiological and physical outcomes, such as the heart rate (HR), intensity, and distance covered, as well as cognitive aspects, including the perception of effort and game enjoyment [1,2]. Therefore, examining the manipulation of the GK’s presence in walking football matches on players’ HR response and distance covered at varying intensities can provide indicators of functional capacity and enjoyment [5]. The exclusion of the goalkeeper was hypothesized to reduce the game intensity, lower the number of shooting actions and avoid the ball being played above the players’ average waist heigh, making walking football more accessible and safer for older adults or those with health conditions [1,2]. This approach aligns with evidence suggesting that lower-intensity formats can encourage sustained participation while minimizing the risk of injury or overexertion [1,2].

Thus, the main objective of this study was to examine the physiological, physical, technical, and perceptual impact of playing walking football WITH-GK vs. WITHOUT-GK. Specifically, we hypothesized that the inclusion of a GK increased the number of shooting actions and consequently the physical intensity and total distance covered, while also elevating players’ HR, particularly in high-intensity zones. Additionally, we hypothesized that playing WITHOUT-GK promoted a passing-oriented style, reducing players’ HR intensity as players adapted to a more possession-driven game. These outcomes were expected to shape the perceived exertion, where we hypothesized higher perceived intensity in the WITH-GK condition and elevated player enjoyment in the WITHOUT-GK setup due to lower exertion and enhanced interaction through passes.

## 2. Materials and Methods

### 2.1. Participants

A total of sixteen (67 ± 4.7 years) male participants were recruited, by convenience sampling, from two local villages in Portugal that habitually play walking football twice a week for ~1 h each session. The inclusion criteria required participants to be older adults (aged > 50) [5] who had been regularly engaging in walking football for at least six months [2]. The exclusion criteria included any recent musculoskeletal injuries, cardiovascular conditions, or other health issues that could interfere with participation in walking football [2]. No dropouts or missing data were registered during data collection. All the participants provided written informed consent prior to participation, and this study was approved by the Portugal Football School, Portuguese Football Federation (CE PFS 01.2024).

### 2.2. Study Design

This study employed a randomized crossover design to assess the impact of playing walking football with and without a GK on physiological, physical, technical, and perceptual variables. Participants engaged in two walking football games under different conditions: one game with a GK (WITH-GK; standard walking football rules were followed, with each team playing with a designated GK) and one without a GK (WITHOUT-GK; the same rules were applied but without the use of GKs). Each participant experienced both conditions in a randomized order (selected randomly by an Excel algorithm), with a one-week washout period between games to prevent eventual fatigue effects.

### 2.3. Methodology

The following walking football rules were defined for both conditions: no running with or without the ball; a maximum of three touches on the ball by player; and no physical contact, including slide tackles [2,5]. Besides these rules, we defined that the ball must always be played below the players’ average waist height [2,5]. The sessions were conducted by a UEFA-certified football coach. For both local villages, participants were organized into two teams (five vs. five players; always maintaining the same five players for each team for each game condition) in two different walking football game conditions, as mentioned above.

Each game condition lasted 30 min, divided into two 15 min halves (with 5 min intervals). The games were played on a standard walking football pitch (30 m × 40 m) in a controlled environment (i.e., indoor wooden pitch) to minimize the external variability. Each game was preceded by a standardized 10 min warm-up consisting of light walking and joint mobility exercises [2].

### 2.4. Physiological Measures

To measure physiological performance during each walking football game, a GARMIN HR (Garmin Ltd., Olathe, KS, USA) monitoring band was used by the participants [8]. The HR was measured for each game condition, with the HRmax initially estimated using the age-based formula (211—0.64 × age) according to Ne, et al.’s [9] equation. However, if players reached HR values during the game that exceeded this predicted maximum, the observed values were instead used as their HRmax. The average HR, % HRmax, and HRpeak were registered. The % Time in Zone 1 (<50%HRmax); % Time in Zone 2 (50–60%HRmax); % Time in Zone 3 (60–70%HRmax); % Time in Zone 4 (70–80%HRmax); % Time in Zone 5 (80–90%HRmax); and % Time in Zone 6 (>90%HRmax) were calculated according to Andersson, Caspers, Godhe, Helge, Eriksen, Fransson, Borjesson and Ekblom-Bak’s [1] HR zones adapted for walking football older participants.

### 2.5. Physical Performance

To measure the physical performance (i.e., total distance covered) during each walking football game for each condition, the WIMU PRO™ Ultra-Wideband (UWB) tracking system (Realtrack Systems, Almeria, Spain) was used. This Local Positioning System (LPS) consists of six UWB antennas, which were arranged outside the participant court, and operates using triangulation between the antennas and the units to derive the X and Y coordinates of each unit [10]. The devices were turned on 10 min before the warm-up and placed on the participants, who wore a customized and specific neoprene vest in the midline between the scapulae at the level of the seventh cervical vertebra (C7). From the beginning to the end of each game condition, the data, excluding the recovery interval (i.e., 5 min), were analyzed using SPRO software (version 989, Realtrack Systems SL, Almeria, Spain). The accuracy and reliability of these devices have been previously reported and validated [11]. The original sampling frequency was 18 Hz. After recording, the X and Y coordinates were smoothed using a 3 Hz Butterworth low-pass filter and resampled to 10 Hz, removing eventual data gaps and synchronizing all the individual data points.

Players’ actions during practice games were video-recorded to integrate technical actions with the spatiotemporal data from the tracking system. Technical actions were captured using LongoMatch, a video notational analysis software, which enabled the precise timing of ball-related events [12,13]. Subsequently, the spatiotemporal data from the tracking system (player positions) and the technical actions from the notational system were combined to integrate the physical, technical, and tactical data. This involved synchronizing player positioning with the notational data and reconstructing the ball’s position through an algorithm. This process allowed for the extraction of technical performance information for comprehensive processing guidelines (refer to [12,13]).

The distances were categorized by speed zones according to Andersson, Caspers, Godhe, Helge, Eriksen, Fransson, Borjesson and Ekblom-Bak [1] and tested for older walking football participants: distance covered at speeds < 4 km/h (low-intensity activity)*;* distance covered at speeds > 4 km/h (higher-intensity walking). Moreover, these parameters were chosen as they are indicative of both volume and intensity [14]. The number of passes, number of shots on goal, number of dribbles, and distance covered while dribbling per player were considered technical performance indicators.

### 2.6. Perceptual Measures

The participants classified the exercise intensity through the rating of the perceived exertion (RPE) using the 10-point OMNI scale (from extremely easy [0 points] to extremely hard [10 points]) at the end of each game condition [15]. In this RPE scale, light intensity was considered 3–4 points, moderate-intensity 5–6 points, and vigorous-intensity 7–8 points [2]. Enjoyment was individually ascertained at the end of all the games conditions through a 5-point Likert scale (1, nothing fun; 2, little fun; 3, indifferent; 4, fun; 5, very fun) with pictograms of human faces [2].

### 2.7. Statistical Analysis

After preliminary inspections for the distribution and assumptions, a paired *t*-test analysis was processed to identify the effect of the game condition (WITH-GK vs. WITHOUT-GK) on the considered variables. The statistical analysis was performed using the Statistical Package for the Social Sciences software (version 24, SPSS, Inc., Chicago, IL, USA), and statistical significance was set at *p* < 0.05.

An estimation techniques approach was used to overcome the shortcomings associated with traditional N-P null hypothesis significance testing [16,17]. Cohen’s *d_unbiased_* (d_unb_) with the 95% confidence interval (CI) as the effect size (ES) (an unbiased estimate has a sampling distribution whose mean equals the population parameter being estimated) was applied to identify pairwise differences between the games [16]. The thresholds for the effect size statistics were 0.2, 0.5, and 0.8 for small, medium, and large [18].

## 3. Results

The descriptive and inferential results of the effect of the game condition on the independent variables are presented in Table 1.

**Figure 1:**
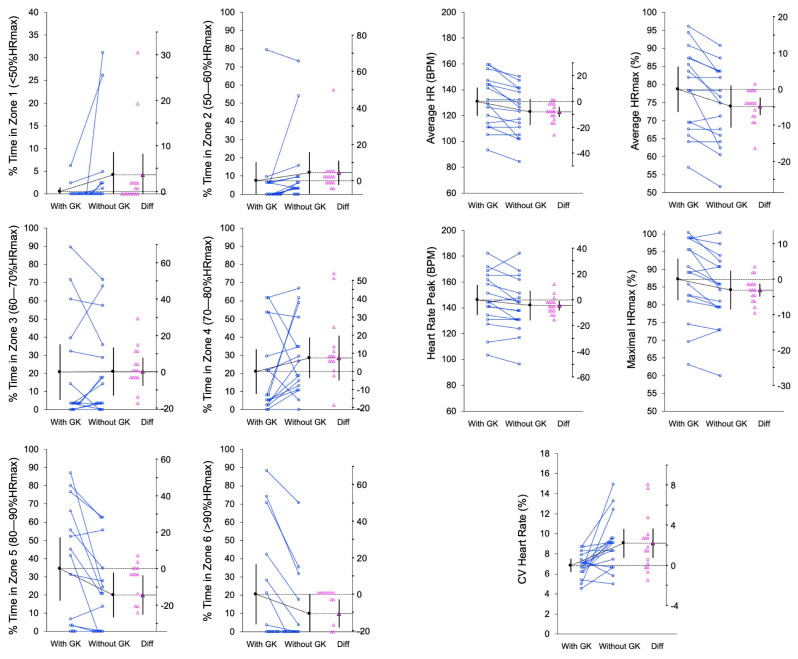
Paired data showing the means and 95% confidence intervals of the WITH-GK and WITHOUT-GK physiological measures. The mean paired difference is shown with its 95% confidence interval against a floating difference axis, whose zero is lined up with the pretest mean. The paired data are shown as small circles joined by lines. The differences are shown as triangles on the difference axis.

**Figure 2:**
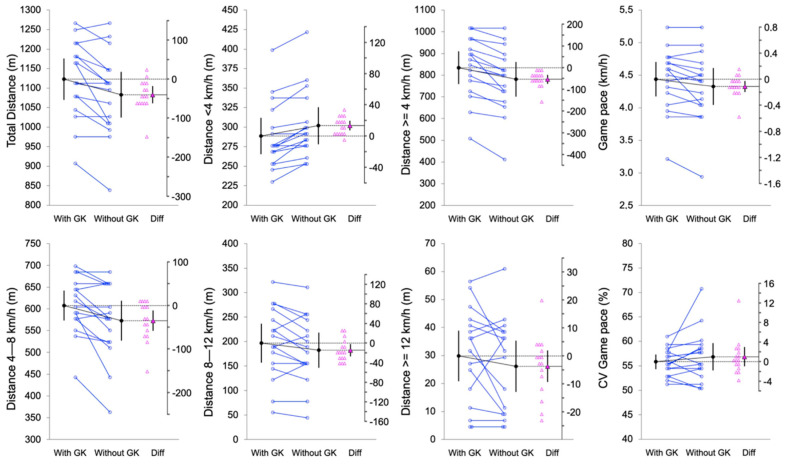
Paired data showing the means and 95% confidence intervals of the WITH-GK and WITHOUT-GK physical performance measures. The mean paired difference is shown with its 95% confidence interval against a floating difference axis, whose zero is lined up with the pretest mean. The paired data are shown as small circles joined by lines. The differences are shown as triangles on the difference axis.

**Figure 3:**
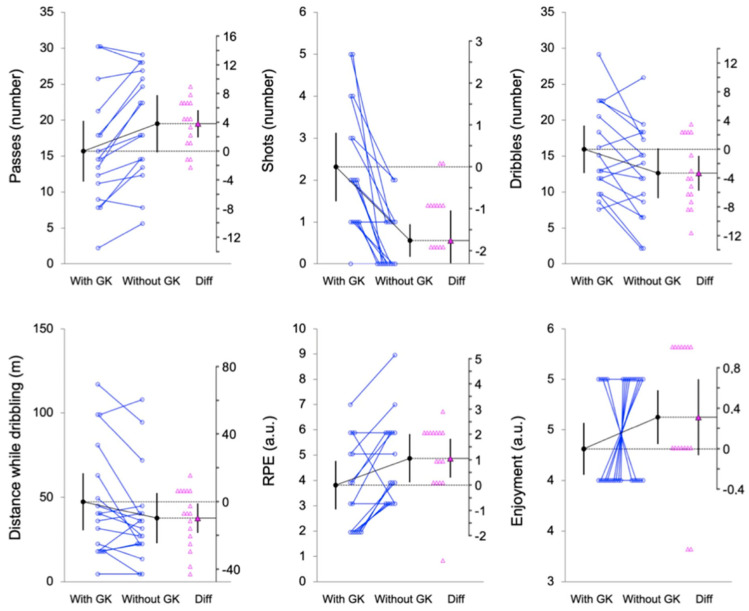
Paired data showing the means and 95% confidence intervals of the WITH--GK and WITHOUT-GK technical and perceptual measures. The mean paired difference is shown with its 95% confidence interval against a floating difference axis, whose zero is lined up with the pretest mean. The paired data are shown as small circles joined by lines. The differences are shown as triangles on the difference axis.

Also, the individual mean values and differences in the mean values from the pairwise comparisons are shown in estimation plots: Figure 1, Figure 2 and Figure 3 for the physical, physiological, and technical and perceptual measures.

**Figure 4:**
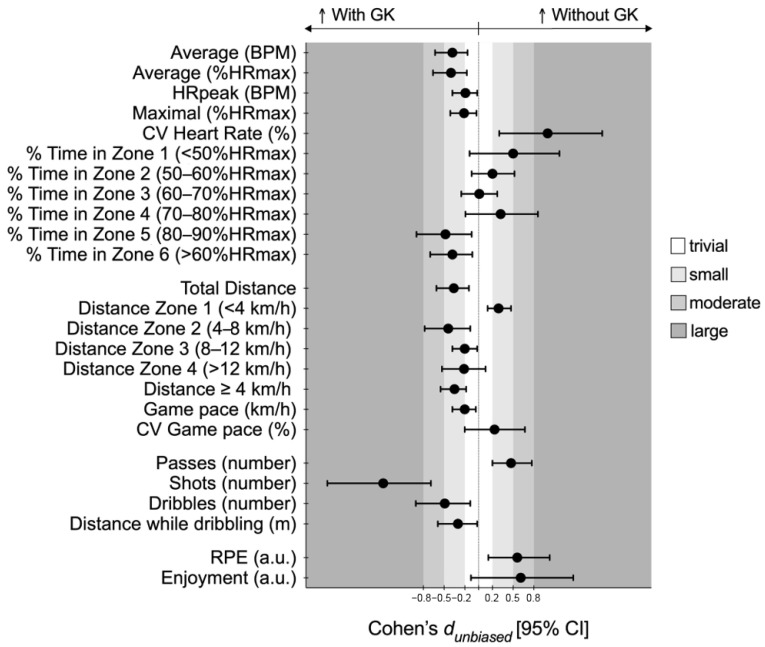
Cohen’s *d_unbiased_* differences for the considered variables according to the game scenarios. Error bars indicate the uncertainty in the true mean changes with the 95% confidence intervals.

Complementarily, the d_unb_ differences with the 95% confidence intervals for all the pairwise comparisons are presented in Figure 4.

In the WITHOUT-GK condition, players exhibited significantly lower values for the HR variables, including the average HR (t = −4.26, *p* < 0.001; d_unb_ = −0.38 [−0.63; −0.16]), average %HRmax (t = −4.24, *p* < 0.001; d_unb_ = −0.40 [−0.66; −0.17]), HRpeak (t = −2.45, *p* = 0.027; d_unb_ = −0.19 [−0.38; −0.02]), and maximal %HRmax (t = −2.48, *p* = 0.025; d_unb_ = −0.21 [−0.41; −0.03]). However, the coefficient of variation showed higher values (t = 3.23, *p* = 0.006; d_unb_ = 1.00 [0.30; 1.79]) in the WITHOUT-GK condition. Regarding the percentage of time spent in different HR zones, players in the WITH-GK condition exhibited increased values in Zone 5 (80–90% HRmax) (t = −2.81, *p* = 0.013; d_unb_ = −0.48 [−0.90; −0.10]) and Zone 6 (>90% HRmax) (t = −2.95, *p* = 0.010; d_unb_ = −0.38 [−0.70; −0.09]).

In terms of the physical performance metrics, players covered significantly greater distances in the WITH-GK condition compared to the WITHOUT-GK condition. This difference was particularly evident for the total distance (t = −3.87, *p* < 0.001; d_unb_ = −0.36 [−0.61, −0.14]) and distance covered at speeds above 4 km/h (t = −5.47, *p* < 0.001; d_unb_ = −0.35 [−0.55, −0.18]). Conversely, the distances covered below 4 km/h were higher in the WITHOUT-GK condition (834.4 ± 141.4 m vs. 781.1 ± 149.8, *p* < 0.001).

For the number of passes, players made significantly fewer passes in the WITH-GK condition compared to the WITHOUT-GK condition, with a mean difference of 3.8 passes per player (95% CI [1.9, 5.6]; t = 4.33, *p* < 0.001; d_unb_ = 0.47 [0.20, 0.77]). Conversely, the number of shots was significantly higher in the WITH-GK condition, with a mean difference of −1.7 shots per player (95% CI [−2.4, −1.0]; t = −5.22, *p* < 0.001; d_unb_ = −1.38 [−2.19, −0.69]). The dribbling actions also differed between conditions, with fewer dribbles observed in the WITHOUT-GK condition (t = −2.92, *p* < 0.01; d_unb_ = −0.49 [−0.91, −0.12]). Additionally, players covered significantly less distance while dribbling in the WITHOUT-GK condition (t = −2.33, *p* = 0.03; d_unb_ = −0.30 [−0.59, −0.02]).

The RPE was significantly higher in the WITHOUT-GK condition (t = 2.96, *p* = 0.01; d_unb_ = 0.56 [0.14, 1.03]). Regarding enjoyment, no significant difference was observed between the conditions, although a positive trend in favor of the WITHOUT-GK condition was identified.

## 4. Discussion

The current preliminary study aimed to investigate the physiological, physical, technical, and perceptual impact of playing walking football WITH-GK vs. WITHOUT-GK. The results indicate that the presence of a GK significantly affects the physiological, physical, technical, and perceptual responses of the participants in walking football matches. Specifically, players who engaged in the WITH-GK condition exhibited a higher average HR and spent more time in elevated HR zones compared to those in the WITHOUT-GK condition, probably due to a reduction of one field player on each team.

The reduction in the HR during the WITHOUT-GK condition could be attributed to the decreased physical engagement required due to low pressure on the ball and more concern about the protection of the goal and prevention of a spatial advantage and counterattacks of the opponent team. In contrast, the WITH-GK condition likely fosters more frequent attacking opportunities and defensive actions, leading to an increased HR and greater physical exertion. Given that, walking football is often played by older adults or individuals looking for a low-impact alternative to traditional football [1]. These findings suggest that using a GK in training or recreational games can increase the intensity based on participants’ fitness levels and objectives.

According to the technical demands of walking football, they can also vary significantly between conditions. In particular, the WITHOUT-GK condition was associated with more passes per player, while the WITH-GK condition saw significantly more shots and dribbling actions. The increase in passing during the WITHOUT-GK condition is likely due to the absence of a central defending figure, affording more ball circulation as players find space to advance without the immediate threat of a GK defending the goal [19]. In addition, the dribbling distance was significantly lower in the WITHOUT-GK condition, suggesting that players are less likely to engage in one-on-one duels or break forward when no GK is present. This could be a result of the overall reduced intensity of the game and the tactical focus shifting toward possession rather than direct attack. It is also important to highlight that the number of shots was significantly higher in the WITH-GK condition, thereby increasing the likelihood of the ball impacting opponents and, as a result, elevating the risk level (i.e., of being safe) of the game. Thus, these findings may emphasize the importance of keeping the game safe and enjoyable by limiting aggressive or high-speed movements [3].

Another finding of this study was the significantly higher ratings of RPE reported in the WITHOUT-GK condition, despite the lower physiological markers of intensity. This could be explained by the nature of walking football, where the pace is generally slower [1], but participants may feel the need to work harder in a more open game without the defensive structure that a GK provides [19]. The psychological effort required to maintain team organization and prevent easy scoring opportunities may lead to increased perceived exertion, even if the physical load is lower. This finding contrasts with studies on traditional football, where higher RPE values typically align with increased physiological demand [20]. In walking football, the slower game pace and emphasis on walking over running likely alter the relationship between physical exertion and subjective fatigue, particularly for older adults [1,2]. The higher perceived exertion in the WITHOUT-GK condition could also reflect the mental fatigue associated with maintaining tactical discipline in a less structured game format [21]. Coaches should consider these perceptual responses when designing training sessions, ensuring that players are not only physically challenged but also mentally engaged, without pushing them toward excessive fatigue. For example, research has shown that elderly participants emphasized the excitement and emotional satisfaction they experienced, as well as the joy of making new friends, which contributed positively to their mental health [4].

Regarding enjoyment, no significant differences were observed between the two game conditions, although there was a trend favoring the WITHOUT-GK condition. This preliminary study provides initial evidence suggesting that the inclusion or exclusion of a goalkeeper does not significantly impact players’ overall enjoyment of the game. Walking football participants often value the social and recreational aspects of the game as much as the competitive elements [3]. Therefore, the choice between playing with or without a GK can be made based on specific training objectives, without detracting from the overall enjoyment of the session.

## 5. Limitations and Future Directions

While this study provides valuable insights into the effects of practice conditions in walking football, several limitations should be noted. First, the sample size was relatively small, which may limit the generalizability of the findings. Future studies should aim to include larger, more diverse populations, including female groups, to explore how these game variations affect a broader range of participants.

Second, the study focused on short-term physiological and technical responses, and it did not assess the long-term impact of repeated exposure to different game conditions on player development. Longitudinal studies could investigate how regularly incorporating WITH-GK and WITHOUT-GK practical sessions into training affects players’ fitness, technical skills, and injury risk over time.

Finally, future research could use qualitative methods, such as player interviews or focus groups, to explore how participants perceive these variations and how they affect their motivation in walking football.

## 6. Practical Applications

The findings of this study offer valuable insights for coaches, trainers, and walking football participants. First, the inclusion or exclusion of a GK can modulate the intensity of walking football sessions.

For older adults or individuals with health conditions, such as cardiovascular disease or reduced mobility, WITHOUT-GK games may offer a safer, lower-intensity option. This format allows players to enjoy the game without excessive physical strain, making it suitable for rehabilitation or injury prevention purposes.

Additionally, coaches can use these game modifications to design training sessions tailored to specific goals, such as improving endurance, passing accuracy, or tactical awareness. Walking football can also be integrated into broader health and wellness programs for aging populations, offering a low impact yet effective way to maintain physical and mental health.

Finally, walking football presents an opportunity to enhance social connections, which are critical for the mental well-being of older adults. The enjoyment scores suggest that both game formats, but especially playing WITHOUT-GK, are satisfying for participants, allowing for flexible training designs that balance physical effort and social interaction.

## 7. Conclusions

This preliminary study highlights that playing WITHOUT-GK provides a safer, lower-intensity alternative to playing WITH-GK, reducing the physical and perceived strain while maintaining enjoyment. The technical impacts, such as increased passing in the WITHOUT-GK condition and more shots in the WITH-GK condition, demonstrate how the configurations influence game dynamics. These findings underscore the need for further research to validate these preliminary insights.

These insights can help coaches and practitioners design more effective training sessions that cater to the unique needs and preferences of walking football participants, particularly older adults. Future research should continue to explore the nuances of walking football to optimize its benefits for health, fitness, and enjoyment.

## Figures and Tables

**Table 1:** Descriptive (mean ± SD) and inferential analysis.

Variables	WITH-GK	WITHOUT-GK	Mean Diff [95% CI]	Paired *t*	*p* Value	Cohen *d_unbiased_* [95% CI]
**Heart Rate**						
Average HR (bpm)	131 ± 21	123 ± 19	−7.97 [−11.96; −3.98]	−4.26	0.001	−0.38 [−0.63; −0.16]
Average HRmax (%)	79 ± 12	74 ± 11	−4.75 [−7.14; −2.36]	−4.24	0.001	−0.40 [−0.66; −0,17]
HRpeak (bpm)	146 ± 22	142 ± 21	−4.42 [−8.26; −0.58]	−2.45	0.027	−0.19 [−0.38; −0.02]
Maximal HRmax (%)	87 ± 11	84 ± 10	−3.03 [−4.91; −1.15]	−2.48	0.025	−0.21 [−0.41; −0.03]
CV Heart Rate (%)	7 ± 1	9 ± 3	2.22 [0.75; 3.68]	3.23	0.006	1.00 [0.30; 1.79]
% Time in Zone 1 (<50%HRmax)	1 ± 2	4 ± 9	3.63 [−1; 8.25]	1.67	0.115	0.50 [−0.13; 1.17]
% Time in Zone 2 (50–60%HRmax)	7 ± 19	13 ± 22	4.4 [−2.27; 11.08]	1.41	0.180	0.20 [−0.10; 0.52]
% Time in Zone 3 (60–70%HRmax)	20 ± 29	23 ± 25	0.26 [−7.48; 7.99]	0.07	0.945	0.01 [−0.25; 0.27]
% Time in Zone 4 (70–80%HRmax)	21 ± 23	29 ± 21	7.53 [−4.74; 19.8]	1.31	0.211	0.32 [−0.19; 0.86]
% Time in Zone 5 (80–90%HRmax)	32 ± 33	22 ± 23	−14.34 [−25.23; −3.45]	−2.81	0.013	−0.48 [−0.90; −0.10]
% Time in Zone 6 (>90%HRmax)	19 ± 31	10 ± 20	−10.54 [−18.15; −2.93]	−2.95	0.010	−0.38 [−0.70; −0.09]
**Distance covered (m)**						
Distance Zone 1 (<4 km/h)	289 ± 44	302 ± 45	13.4 [7.2, 19.5]	4.63	<0.001	0.29 [0.13, 0.47]
Distance Zone 2 (4–8 km/h)	608 ± 65	573 ± 86	−34.9 [−58.6, −11.3]	−3.15	<0.01	−0.44 [−0.78, −0.12]
Distance Zone 3 (8–12 km/h)	197 ± 75	182 ± 67	−14.6 [−27.1, −2.0]	−2.48	0.03	−0.20 [−0.38, −0.02]
Distance Zone 4 (>12 km/h)	30 ± 17	26 ± 17	−3.7 [−9.3, 1.9]	−1.40	0.18	−0.21 [−0.53, 0.10]
Distance ≥ 4 km/h	834 ± 141	781 ± 150	−53.2 [−74.0, −32.5]	−5.47	<0.001	−0.35 [−0.55, −0.18]
Total Distance	1123 ± 99	1083 ± 110	−39.8 [−61.8, −17.9]	−3.87	<0.001	−0.36 [−0.61, −0.14]
Game pace (km/h)	4 ± 1	4 ± 1	−0.1 [−0.2, −0.1]	−2.77	0.01	−0.20 [−0.38, −0.04]
CV Game pace (%)	56 ± 3	57 ± 5	1.0 [−0.9, 2.8]	1.10	0.29	0.23 [−0.20, 0.67]
Passes (number)	16 ± 8	20 ± 8	3.8 [1.9, 5.6]	4.33	<0.001	0.47 [0.20, 0.77]
Shots (number)	2 ± 1	1 ± 1	−1.7 [−2.4, −1.0]	−5.22	<0.001	−1.38 [−2.19, −0.69]
Dribbles (number)	16 ± 6	13 ± 7	−3.3 [−5.7, −0.8]	−2.92	<0.01	−0.49 [−0.91, −0.12]
Distance while dribbling (m)	47 ± 33	38 ± 29	−9.6 [−18.8, −0.8]	−2.33	0.03	−0.30 [−0.59, −0.02]
RPE (a.u.)	4 ± 2	5 ± 2	1.0 [0.3, 1.8]	2.96	0.01	0.56 [0.14, 1.03]
Enjoyment (a.u.)	4 ± 1	5 ± 1	0.3 [−0.1, 0.6]	1.78	0.10	0.61 [−0.11, 1.37]

Abbreviations: HR, heart rate; CV, coefficient of variation; RPE, perceived effort of exertion.

## Data Availability

Data supporting the findings of this research are available from the corresponding author (Júlio A. Costa) upon reasonable request.
